# Green synthesis and characterization of Fe_3_O_4_ nanoparticles using *Chlorella*-K01 extract for potential enhancement of plant growth stimulating and antifungal activity

**DOI:** 10.1038/s41598-021-01538-2

**Published:** 2021-11-09

**Authors:** Theint Theint Win, Sikandar Khan, Bo Bo, Shah Zada, PengCheng Fu

**Affiliations:** 1 State Key Laboratory of Marine Resource Utilization in South China Sea, Hainan University, 58 Renmin Avenue, Haikou, 570228 Hainan Province China; 2 Biotechnology Research Department, Ministry of Science and Technology, Kyaukse, 05151 Myanmar; 3 Department of Biotechnology, Shaheed Benazir Bhutto University, Sheringal, KP 18000 Pakistan; 4 Beijing Key Laboratory for Bioengineering and Sensing Technology, Research Center for Bioengineering and Sensing Technology, School of Chemistry and Bioengineering, University of Science and Technology, Beijing, 100083 China

**Keywords:** Biotechnology, Materials science

## Abstract

The purpose of this research was to determine the efficacy of iron oxide nanoparticles (Fe_3_O_4_-NPs) using microalgal products as a plant growth stimulant and antifungal agent. The work was conducted with the phyco-synthesis and characterization of Fe_3_O_4_-NPs using 0.1 M ferric/ferrous chloride solution (2:1 ratio; 65 °C) with aqueous extract of the green microalga *Chlorella* K01. Protein, carbohydrate and polyphenol contents of *Chlorella* K01 extract were measured. The synthesized microalgal Fe_3_O_4_-NPs made a significant contribution to the germination and vigor index of rice, maize, mustard, green grams, and watermelons. Fe_3_O_4_-NPs also exhibited antifungal activity against *Fusarium oxysporum, Fusarium tricinctum, Fusarium maniliforme, Rhizoctonia solani,* and *Phythium* sp. Fourier transform infrared spectroscopy (FTIR), X-ray diffraction (XRD), X-ray photoelectron spectroscopy (XPS) scanning electron microscopy (SEM), transmission electron microscopy (TEM), particle size analysers (PSA), and zeta potential (ZP) measurements were used to characterize these green fabricated magnetite NPs. FTIR analysis showed that the synergy of microalgal proteins, carbohydrtates and polyphenols is responsible for the biofabrication of iron nanoparticles. A spheroid dispersion of biosynthesized Fe_3_O_4_-NPs with an average diameter of 76.5 nm was produced in the synthetic process.

## Introduction

Iron (Fe) is a nutrient that is required by all life forms, yet its deficiency is prevalent in many different crops^1,2^. Iron plays a variety of important roles in plants, including the biosynthesis of chlorophyll, respiration, and the regulation of redox reactions^3,4^. Crops growing on soil that are Fe-deficient lead to crops that are Fe-deficient which reduces yield and quality^5^. On the other hand, in perspective of the food chain, Fe deficiency can lead to anaemia in living creatures^6^. In order to overcome this problem, inorganic-iron, chelated-iron fertilizer, and organic-iron, are currently used^7^. High cost and its absorption potential^8,9^ are drawback of currently used fertilizers, therefore, tformulation of using Fe fertilizer need to be improved.

Recent advances in the study of biofertilizers in the field of algae biotechnology have been made. *Chlorella* sp. is widely accepted as a model microorganism for academic studies^10–13^. Using algae-based biofertilizers, which offer substantial benefits to green agriculture, has proven advantageous, with three key goals: healthy environment, economic profits, and socioeconomic equity. These can be met through the applications of algal biofertilizers in sustainable agriculture^14^. Algal metabolites have been found to promote mineralization and plant–microbe symbiosis by providing nutrients to the soil microbial community^15^. Furthermore, addition of cyanobacterial filtrates to plant seeds is een to increase the average germination^16^. Biogenic nanoparticles have proven to be effective nanomaterial-based fungicides for the control of some plant fungal diseases^17^. They have the potential to be widely used in agriculture as biocontrol agents to promote sustainable agriculture^18^. The environmentally green chemistry approach thus provides a clean, nontoxic, and environmentally friendly method of producing NPs with a wide range of size, morphology, component, and physical and chemical properties^19^. Moreover, metal oxide nanoparticles are stable and are considered to be safe for humans^20^.

The main purpose of this study was to synthesize iron (Fe) nanoparticles based on microalgae for agricultural purposes. Aqueous extracts of *Chlorella* K01 have been used to biosynthesize environmenta friendly plant growth stimulants and anti-fungal Fe nanoparticles (Fe_3_O_4_-NPs).

## Materials and methods

All chemicals used were of analytical reagent grade and purchased from Aladdin, China. *Chlorella* K01 was gifted by Professor Prezemyslaw Malec and Dr. Jan Burczyk from Jagiellonian University, Krakow, Poland.

### Preparation of algal extract and determination of protein, carbohydrate and polyphenol contents

*Chlorella* K01 was cultivated in fresh KC medium at 25–40 (μE m^−2^ s^−1^). Start-up seed culture was done in 250 ml sterile flasks having 100 ml KC medium. To obtain biomass, it was subcultured in 500 ml flasks and then larger 1000 ml flasks. The culture was washed with water, freeze dried and stored. To prepare this extract, 100 ml of ultrapure water was heated at 60–70 °C with 0.1 g of algal powder. The raw extract algal was constantly agitated, then filtered and the obtained supernatant was used as the algal bioextract.

The kit #16-6002 Bao Ruyi (Beijing) Biotechnology Co., Ltd used the BCA method to determine protein concentrations (bicinchoninic acid)^21^. The BCA/copper complex absorbance was measured at 562 nm in a UV–Vis spectrophotometer. A 0.5 mg ml^−1^ bovine serum albumin standard curve was used to calculate protein concentration. The total phenolic content of the extracts calculated by using the Folin–Ciocalteu method^22^, gallic acid was the standard. The carbohydrate content was estimated using the phenol–sulfuric acid method. The absorbance at 490 nm was measured the colored aromatic complex formed between the phenol and the carbohydrate with glucose as a standard.

### Synthesis of ***Chlorella*** K01 based Fe_3_O_4_ NPs

0.1 M FeCl_2_·4H_2_O and 0.1% algal extract were added in the ratio 2:3, under four different pH conditions. NaOH was used to adjust the pH to 6, 8, 10, and 12. These reactions were kept at 60–70 °C. The synthesized Fe_3_O_4_-NPs were washed three times with 70% ethanol and dried in a hot air oven for 24 h. These iron oxide NPs were synthesized and stored until further use. The effects of germination on various Fe_3_O_4_-NPs seeds synthesized at different pH levels have been examined as shown in “Seed treatment and in vitro seed germination test” section.

### Seed treatment and in vitro seed germination test

The commercialized seeds (rice, maize, mustard, green grams, and watermelons) were bought from the local Longhua market (20° 2′ 9.672″ N, 110° 20′ 3.2634″ E). All the seeds tested in the research are permitted and legal for trade, commercialization in China. Therefore Specific permission was not needed from the Local Authority.

Germination at various Fe_3_O_4_-NPs concentrations was evaluated to determine plant toxicity, as described by Stampoulis et al.^23^. Firstly, soak the seeds in 0.1% mercuric chloride for 3 min, and then rinse thrice with sterilized distilled water. The aseptic seeds were soaked in solution containing (synthesized nanoparticles 1 mg ml^−1^, 5 mg ml^−1^, 7 mg ml^−1^, 10 mg ml^−1^, bulk FeCl_2_·4H_2_O (0.1 M) Gibberellic acid (GA) 15 mg ml^−1^) for 1 h and agitated at 100 rpm. The control was sterilized and treated with distilled water. Afterwards, all of the seeds were transferred to plates that contained two layers of wetted filter papers that were carefully rolled (25 seeds per plate) and incubated at 25 °C under a 16:8 (light: dark) cycle for 7 days. The germination percentage was calculated using normal seedlings. Ten seedlings from each replicate were chosen at random to be measured for shoot and root length. Triplicate experiment was repeated three times. For their comparative study, the vigor index was calculated for seeds throughout germination experiments that exhibit the best responses to Fe_3_O_4_, bulk FeCl_2_·4H_2_O (0.1 M), Gibberellic acid, and control. A vigor index was calculated as given by Abdul-Baki and Anderson^24^:

$${\text{Vigor}}\;{\text{index}}\;{\text{I}} = {\text{Germination}}\;\% \times {\text{Seedling}}\;{\text{length}}\;\left( {{\text{Root}} + {\text{Shoot}}} \right)^{25}$$

### Anti-fungal activity against phytopathogens

The antifungal activity of Fe_3_O_4_-NPs was examined against the fungal pathogens; *Fusarium oxysporum, Fusarium tricinctum, Fusarium maniliforme, Rhizoctonia solani,* and *Phythium* sp. Fungal cultures were incubated in potato dextrose media (PDA) liquid media at 26 °C for 5 days before being cultured on fresh PDA solid media with 1 × 10^7^ spores ml^−1^. The agar well diffusion method^25^ was used in this study. PDA medium was punched into 8 mm wells and 100 μl of Fe_3_O_4_-NPs at 1 mg ml^−1^ were added. The plates were incubated at 26 °C for 5 days. For the experimental control, plates containing no Fe_3_O_4_-NPs were incubated under same conditions.

### Characterization

The synthesized nanoparticles were characterized via, Fourier Transformed Infrared Spectroscopy (FT-IR), X-ray Diffraction (XRD), X-ray photoelectron spectroscopy (XPS), Zeta potential, Dynamic light scattering (DLS), scanning electron microscope (SEM), and transmission electron microscopes (TEM).

#### Electron microscopy

On the carbon-coated copper grids, a drop of Fe_3_O_4_-NPs colloid (50 μl) was placed. TEM was used to examine the morphology and size of phytogenic Fe_3_O_4_-NPs (TEM). TEM micrographs have been captured through the analysis of the prepared grids on an AMT camera system. The particle size was also determined by TEM using an Image Analyser System (IAS). The surface features of Fe_3_O_4_-NPs deposited on a graphite grid were examined using a SEM (model S360 brand SEM—Leica Cambridge, Cambridge, UK).

#### X-ray diffraction (XRD) and X-ray photoelectron spectroscopy

Cu Kα radiation was used to generate the x-ray diffraction (XRD) pattern, which was recorded using an X-ray diffractometer (Powder X-ray-D8 advanced diffractometer, Burker) from 5_ to 100_ 2θ angles at 40 kV and 30 mA. The exposure time was 300 s. Besides, X-ray photoelectron spectroscopy (Thermo Scientific ESCALAB 250) was used to further analyse the chemical composition and binding energies of the as prepared Fe_3_O_4_ nanoparticles^26^.

#### Fourier transform infrared (FTIR) spectral analyses

The Fe_3_O_4_-NPs colloid was biosynthesized and centrifuged at 10,000 g for 15 min after being lyophilized and grinded with potassium bromide (KBr) powder for FTIR measurements. The spectrum was captured in the 500–4000 cm^–1^ range using a Bruker, TGA-IR, TENSOR 27 spectrometer in diffuse reflectance mode with a resolution of 4 cm. In accordance with previously published information, spectral absorption bands were identified.

#### Zeta potential and dynamic light scattering (DLS)

With the help of dynamic light scattering (DLS) and zeta potential (HORIBA Zetasizer SZ-100), the distribution and size of Fe_3_O_4_-NPs were determined.

### Statistical analysis

Triplicated data of each experiment was analysed statistically using one-way analysis of variance (ANOVA); Minitab 17.1.0 (Minitab Pty, Sydney, Australia) and Excel software, and the mean values for each treatment were compared using the Turkey's test at the *P* < 0.05 confidence level.

## Results and discussion

**Figure 1:**
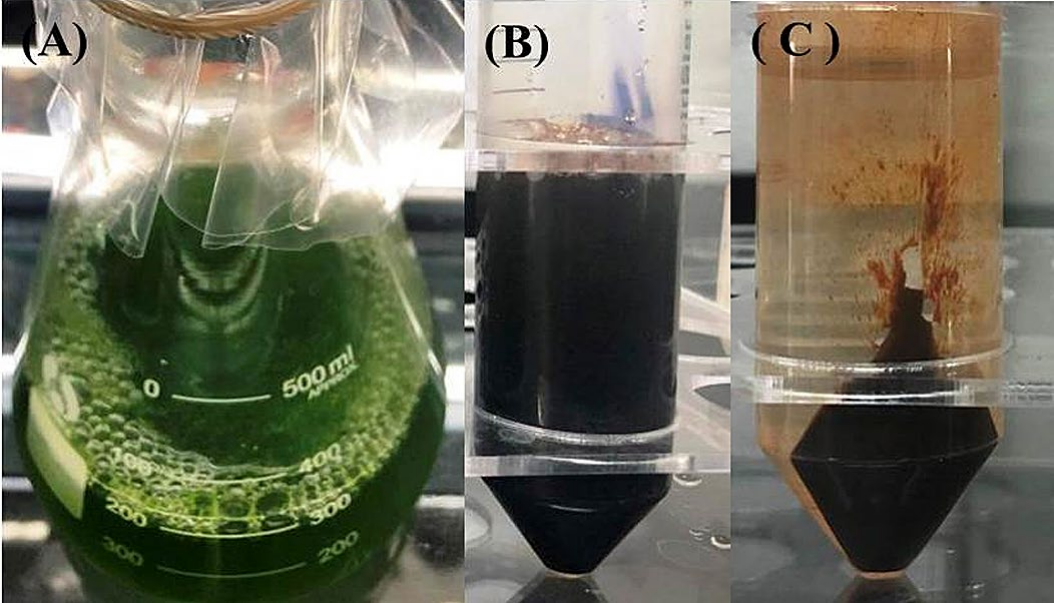
One month old *Chlorella* K01 culture (**A**) and synthesized Fe_3_O_4_-NPs solution (**B**), Crude Fe_3_O_4_-NPs (**C**).

Iron oxide nanoparticles were successfully prepared using a green approach with microalgal extract in an alkaline medium. Fe_3_O_4_-NPs was created using a microalgal extract in a quick, cost-effective, and environmentally safe way^19^. According to El-Kassas et al. (2017), proteins and polyphenols from *Chlorella* K01 catalyse the reduction of iron ions into nanoparticles, and polysaccharides stabilize Fe_3_O_4_-NPs^27^. *Chlorella* K01 extract contains 632 ± 195 mg ml^−1^ of protein, 39.59 ± 3.04 mg ml^−1^ of carbohydrate and 0.12 ± 0.007% of polyphenol content. Therefore, the extract can be used for the bio fabrication of Fe_3_O_4_-NPs. 1 g of dry algal powder yielded 16 mg at pH-6, 645 mg at pH-8, 703 mg at pH-10 and 829 mg at pH-12 of Fe_3_O_4_-NPs. Figure 1 illustrates one month old *Chlorella* K01 culture, aqueous Fe_3_O_4_-NPs solution and Crude Fe_3_O_4_-NPs.

**Figure 2:**
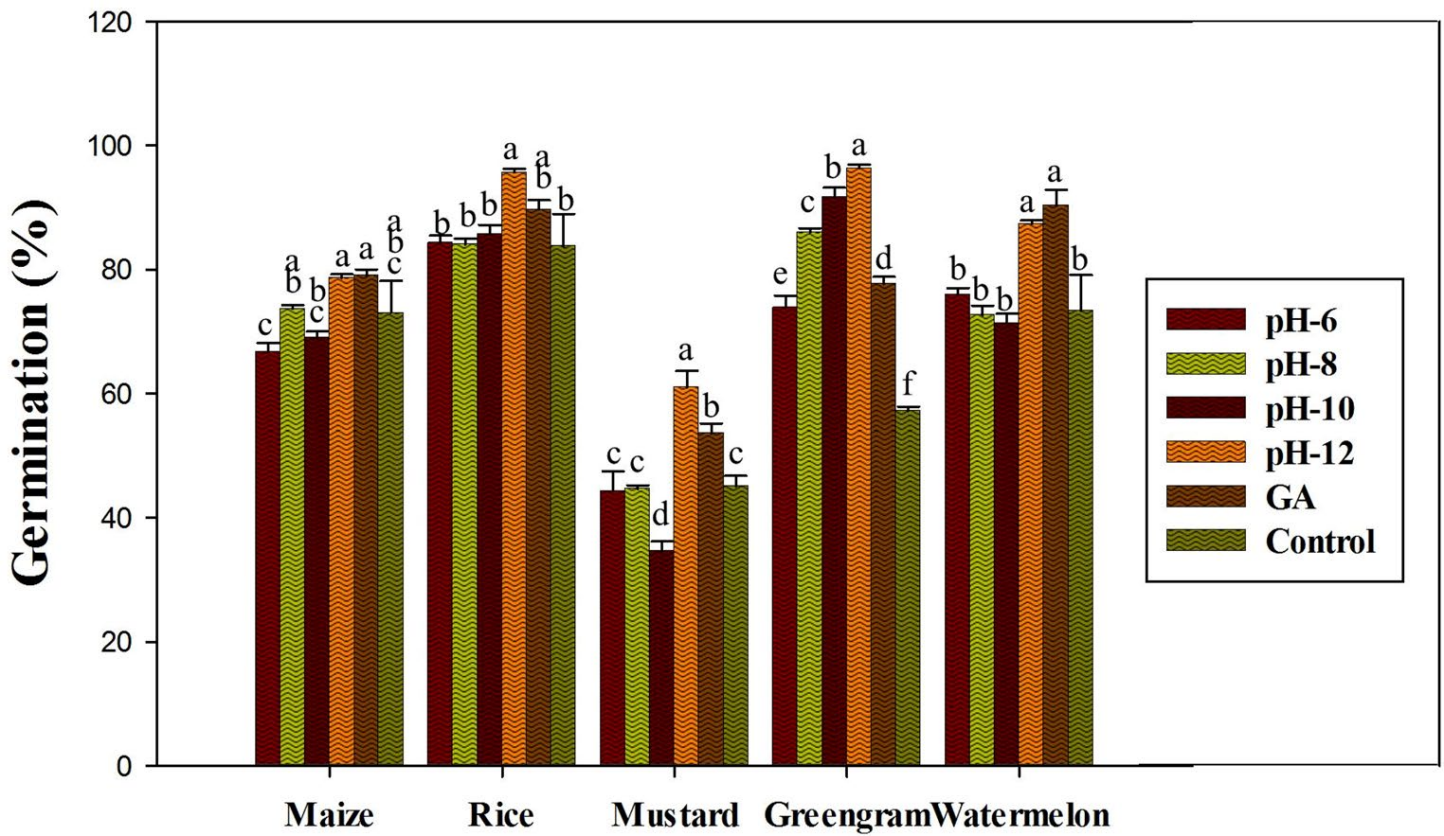
Germination of maize, rice, mustard, greengram and watermelon with different Fe_3_O_4_-NPs synthesized by different pH. Data are mean ± SD. Each group without sharing letter mean statistical significance (*P* ≤ 0.05).

**Figure 3:**
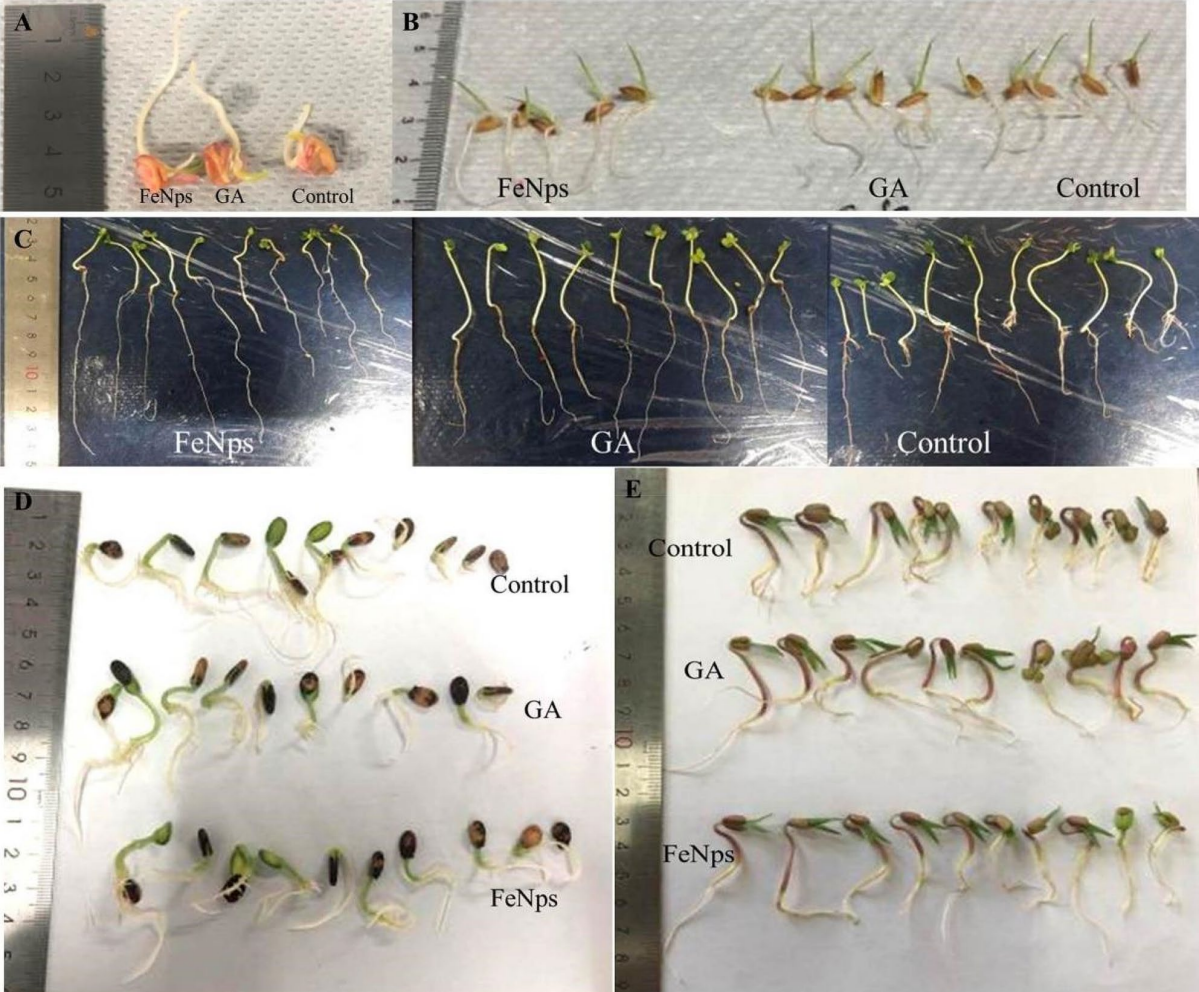
Germination characteristics of tested crops (**A**) corn, (**B**) rice, (**C**) mustard, (**D**) watermelon, (**E**) greengram.

**Figure 4:**
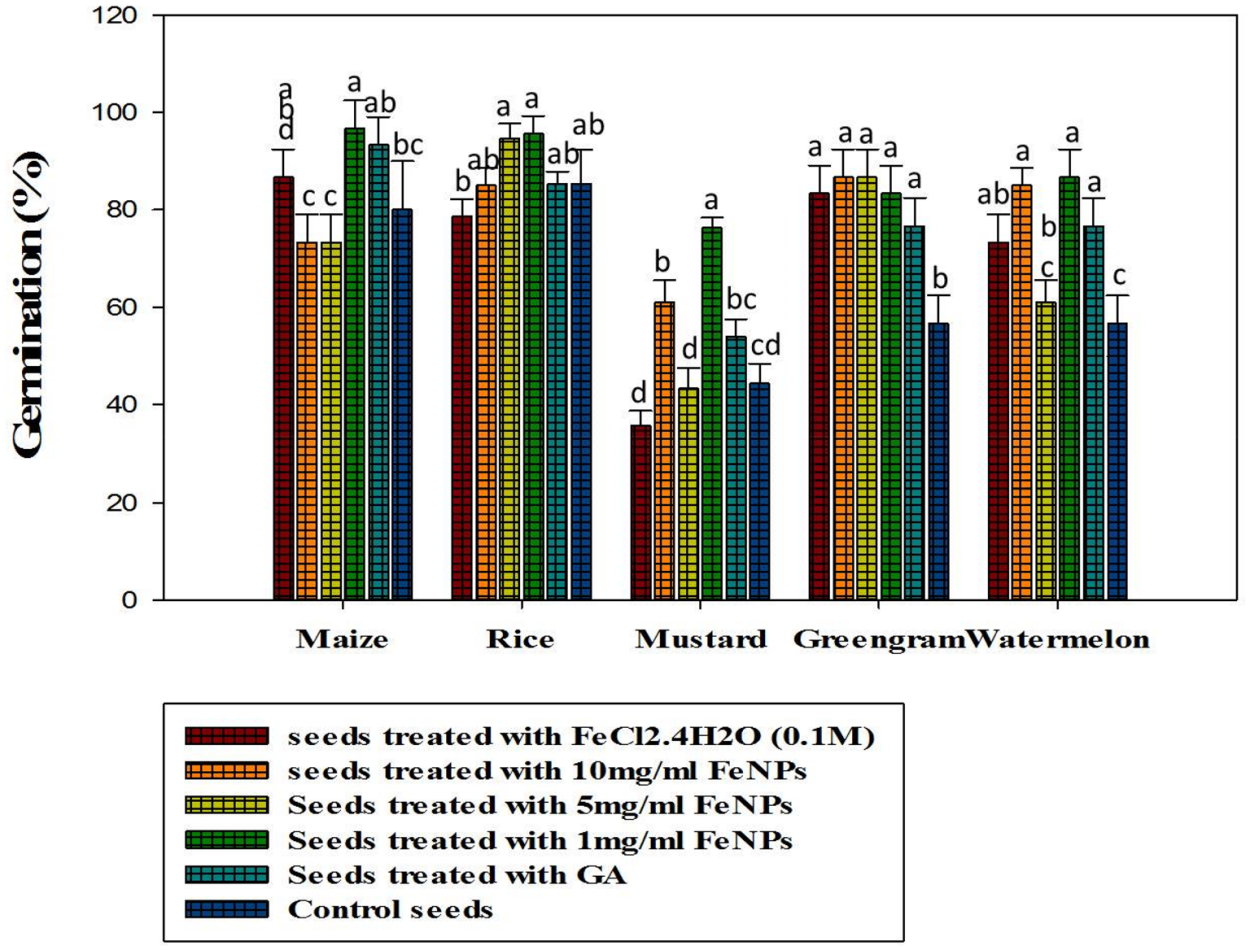
Percentage of seed germination in vitro condition with different doses of Fe_3_O_4_-NPs synthesized by pH-12. Data are mean ± SD. Each group without sharing letter mean statistical significance (*P* ≤ 0.05).

**Figure 5:**
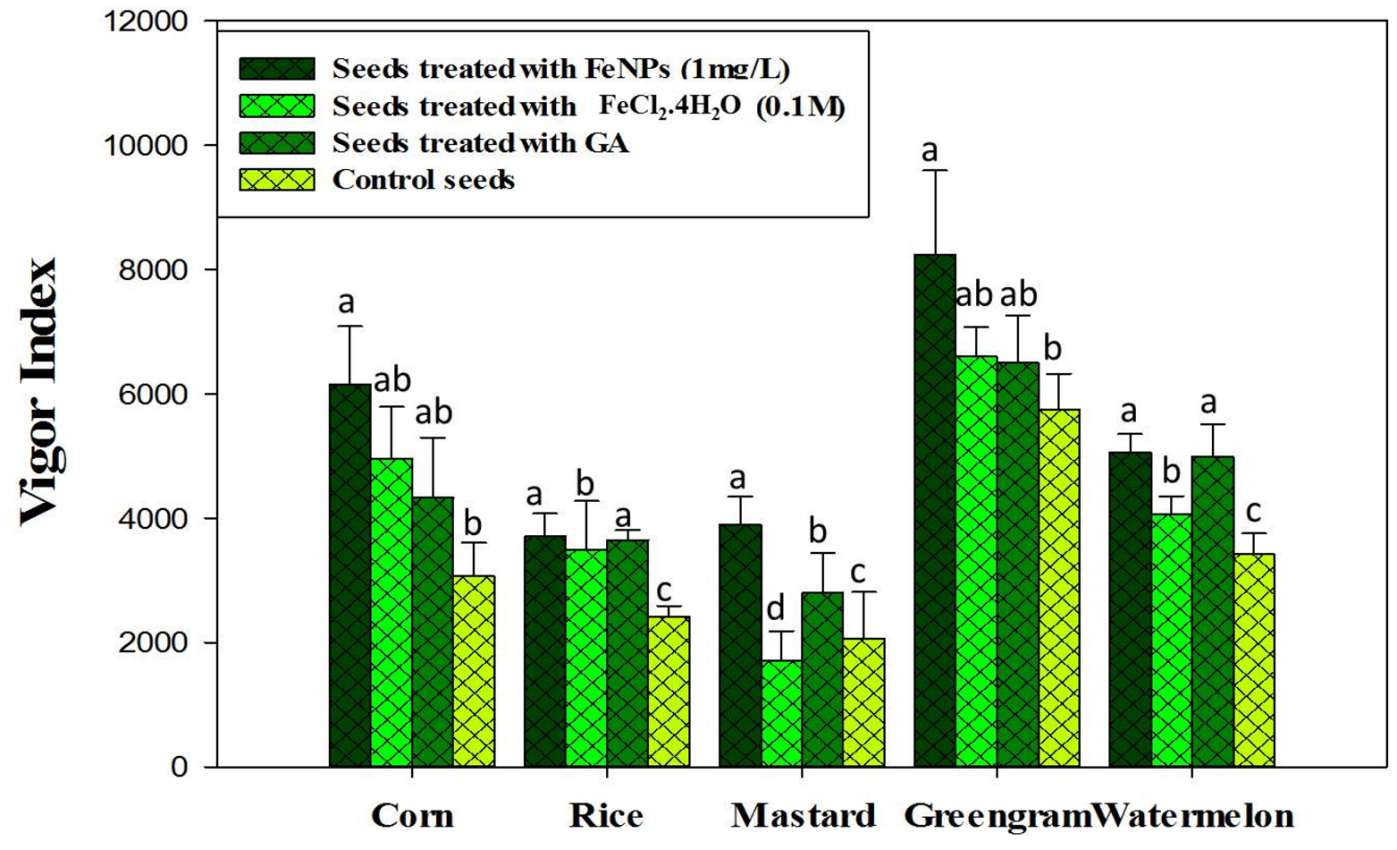
Effect of Fe_3_O_4_-NPs, bulk FeCl_2_.4H_2_O (0.1 M), and Gibberellic acid on Vigor index of the tested crops. Values in each bar are represented as mean ± SD.

According to the results of the in vitro germination test, Fe_3_O_4_-NPs synthesized at pH-12 showed significantly higher germination rates (*P* ≤ 0.05) and was therefore used for further investigation (Fig. 2). The description of the crops used in this experiment, as well as their germination activity, is shown in Fig. 3. In terms of the effect of different Fe_3_O_4_-NPs concentrations on seed germination, Fe_3_O_4_-NP-treated seeds (1 mg ml^−1^) had a higher germination rate, a higher vigor index, and a notable increase in seedling shoot and root formations (*P* ≤ 0.05) among the crops than GA treated seeds and control seed (Figs. 4, 5). When compared to the positive and negative control seedlings, the Fe_3_O_4_-NPs treatments had a higher germination rate, root length, and vigor index (*P* ≤ 0.05). Among the crops tested, green gram demonstrated the most remarkable plant growth and vigor index (Figs. 3, 5). Fe nanoparticles have been shown to have a negative effect on the germination process and germination parameters of sunflower seedlings^25^. Few reports on the effects of Fe-NPs in plants are available, Shankramma et al.^28^ stated that the research outcomes depend on the nature of the NPs and plant species and are not always consistent with each other. However, the crops in this study showed no negative effects during germination with varying concentrations of Fe_3_O_4_-NPs. This is due to the reaction of plant growth stimulant microalgae metabolites with FeCL_2_^14^. This is consistent with the findings of Ilona et al. (2019), who found that Fe_3_O_4_-NPs at concentrations of 1 mg L^−1^, 2 mg L^−1^, and 4 mg L^−1^ induce low genotoxicity and have a beneficial effect on the growth and development of rocket seedlings, implying that nanoparticles may improve plants' resistance to environmental stresses^29^. Polischuk et al. (2019) also noted that Fe_3_O_4_-NPs treatment increased 10% in seed germination compared to the control, as well as a 25–30% increase in root growth^30^. On the other hand, González-Melendi et al. (2008) and Zhu et al. (2008) searched into Fe-oxides and found that they are relatively safe for nanoparticle delivery in plants^31,32^.

**Figure 6:**
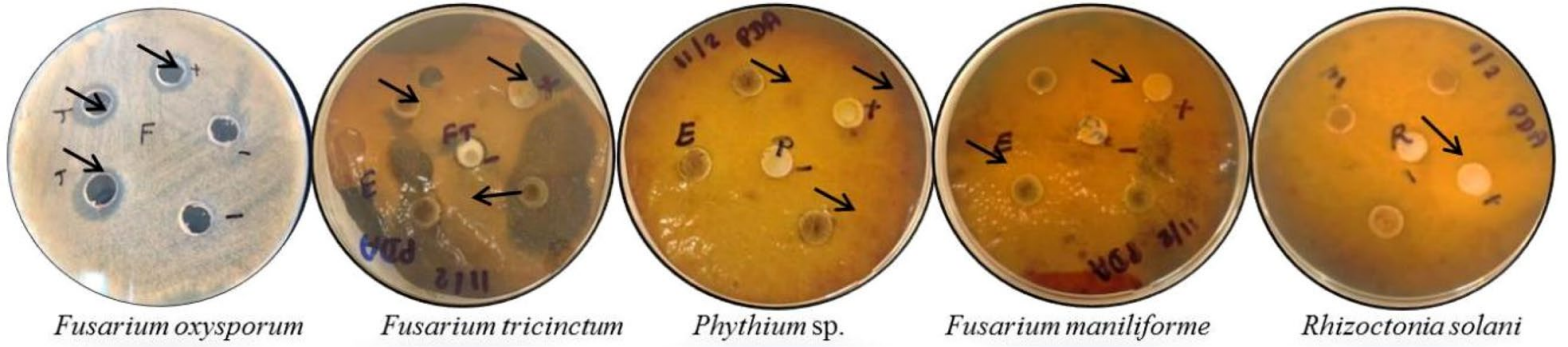
Antifungal zone of inhibition by iron oxide (Fe_3_O_4_) nanoparticles (From left to right) *Fusarium oxysporum, Fusarium tricinctum, Phythium* sp. *Fusarium maniliforme,* and *Rhizoctonia solani*.

Qualitative assessment of antifungal activity against *Fusarium oxysporum, Fusarium tricinctum, Fusarium maniliforme, Rhizoctonia solani,* and *Phythium* sp. growth were carried out. All tested fungal growth showed inhibition when treated with Fe_3_O_4_-NPs. Each phytopathogen had an inhibition zone diameter ranging from 10 to 25 mm (Fig. 6). The results clearly demonstrate that iron oxide nanoparticles at the concentrations used in this study (1 mg L^−1^) showed the inhibition of radial growth of all fungal pathogens tested. The appearance of an inhibition zone on culture media demonstrates the iron oxide nanoparticles' biocidal activity^33^. Additionally, Nehra et al. (2017) demonstrated that iron oxide nanoparticles have antifungal and antibacterial activity. As a result, they concluded that iron oxide nanoparticles can be effectively used as antimicrobial agents^34^.

**Figure 7:**
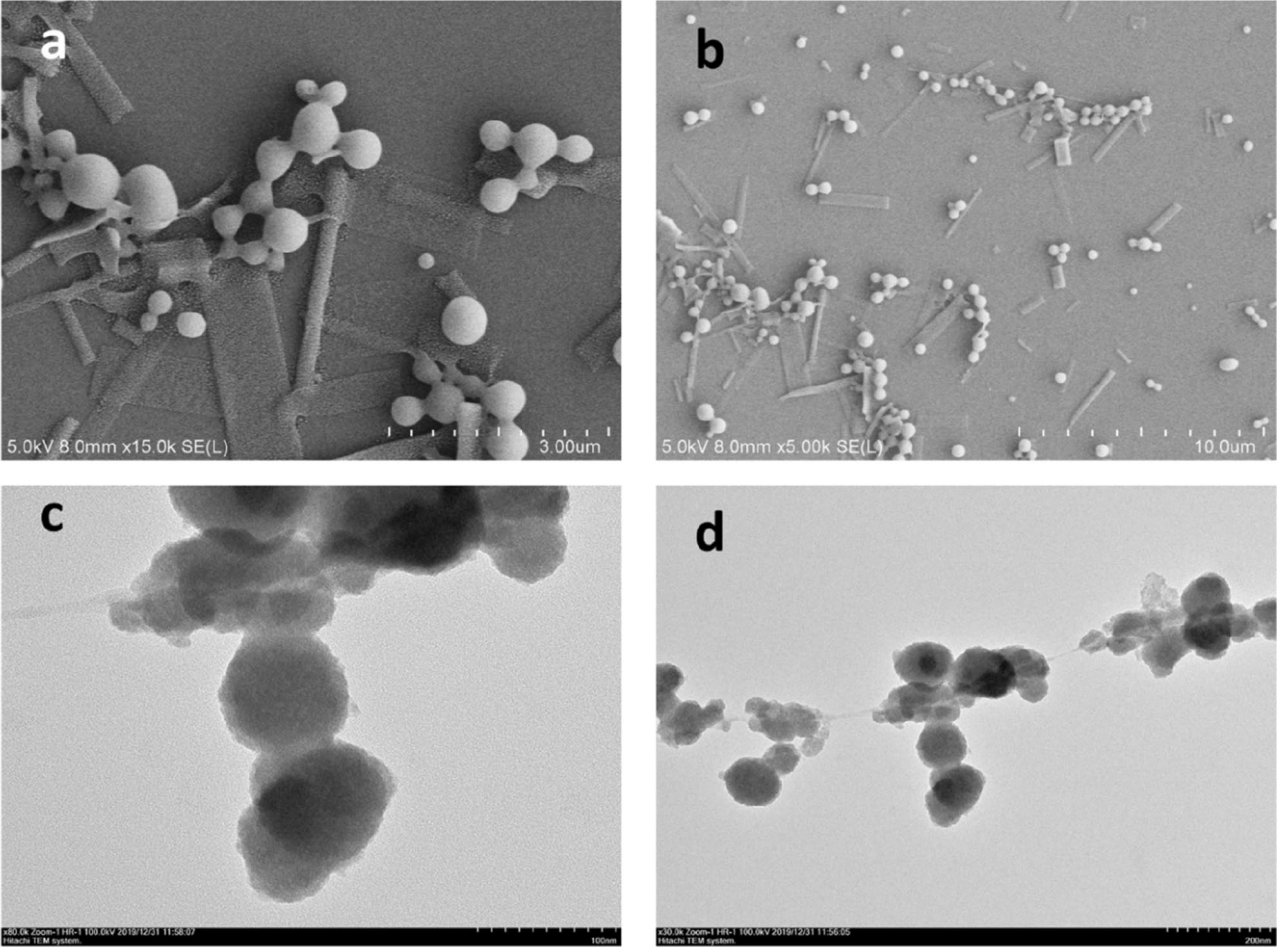
SEM (“**a**” and “**b**”) and TEM (“**c**” and “**d**”) images of the as prepared Fe_3_O_4_-NPs using *Chlorella* K01 extracts as capping and reducing gents.

Morphological study of Fe_3_O_4_-NPs was conducted using both the scanning and transmission electron microscopy (Fig. 7). It can be observed in the SEM images that the *Chlorella* K01 extracts mediated synthesis of Fe_3_O_4_-NPs in a monodispersed form that are spherical in shape, and which are well separated without any evident aggregation (Fig. 7a,b). This excellent dispersion and spherical morphology of the NPs can be ascribed to the outstanding capping ability of the biochemical in the extracts of *Chlorella* K01. TEM analysis revealed the size and morphology of the synthesized NPs. The spherical biofabricated Fe_3_O_4_-NPs were in the range of approximately 50 to 100 nm in size (Fig. 7c,d).

**Figure 8:**
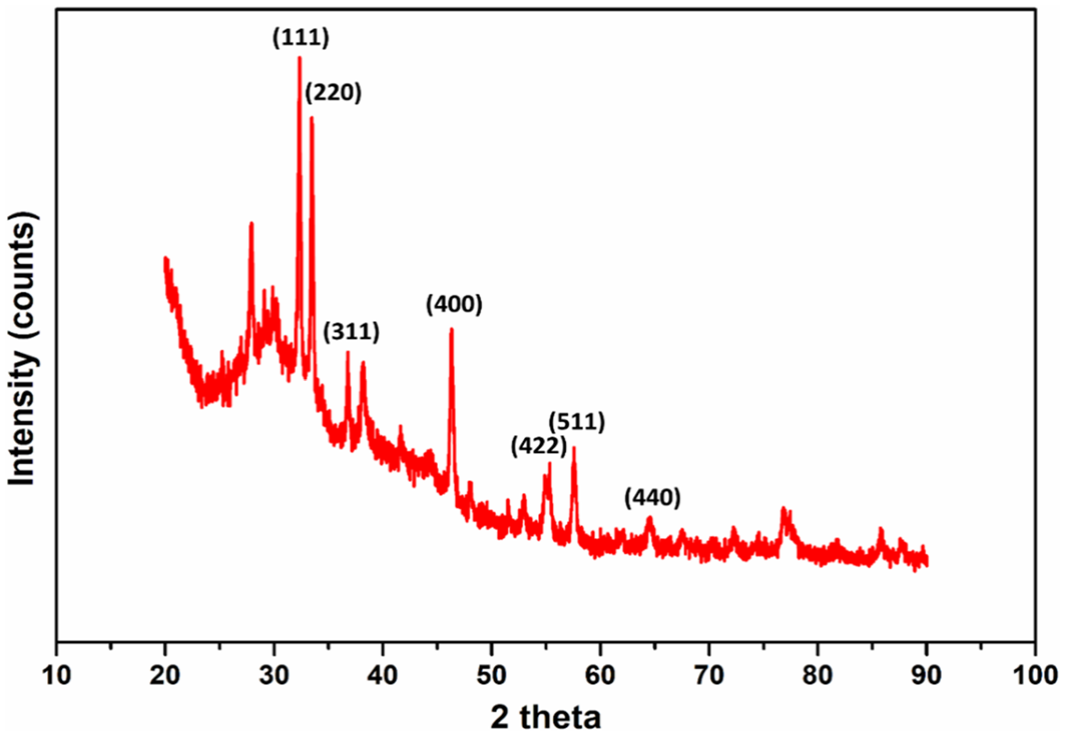
XRD pattern of the biosynthesized Fe_3_O_4_-NPs.

By employing an X-ray diffraction technique, we were able to determine the crystalline structure of the biofabricated Fe_3_O_4_-NPs. The XRD profile of the biofabricated Fe_3_O_4_-NPs is illustrated in Fig. 8. The XRD pattern of the Fe_3_O_4_-NPs depict various spectral peaks at 2-theta = 31.2°, 33.6°, 36.2°, 46°, 54.8°, 57.1°, and 64°, that can be ascribed to their relevant indices and diffraction planes (111), (220), (311), (400), (422), (511), and (440), respectively. The diffraction planes of the current Fe_3_O_4_-NPs are very much similar to that reported earlier for Fe_3_O_4_-NPs^35^.

**Figure 9:**
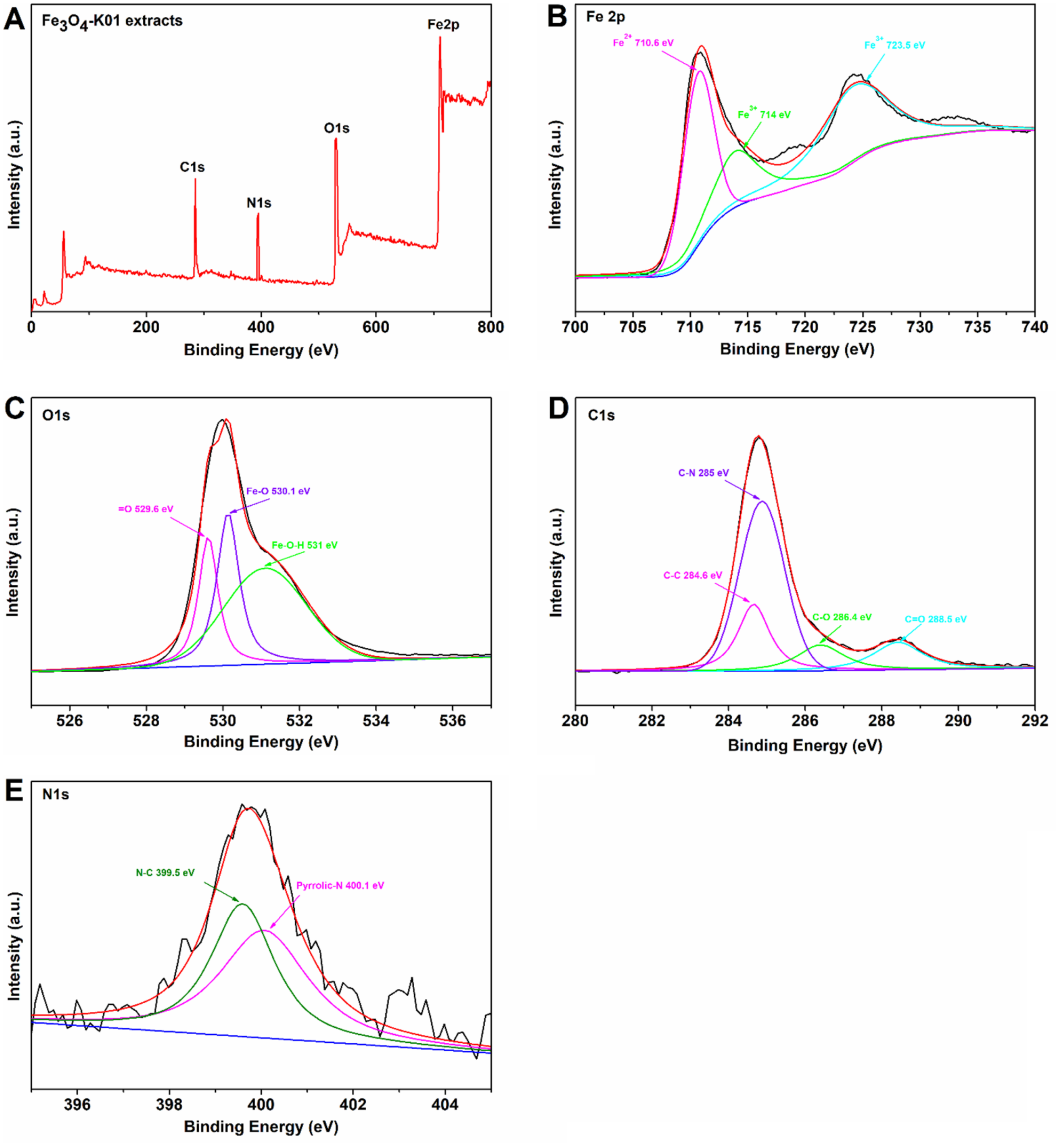
X-ray photoelectron spectra (XPS) of Fe_3_O_4_-K01 extracts. Survey scan XPS spectrum (**A**), Fe 2p (**B**) O1s (**C**), C1s (**D**) and N1s (**E**) spectra of the K01 extracts based synthesis of Fe_3_O_4_ nanoparticles. The black lines (little noisy) denote experimental raw data, the overlaid red line is the deconvoluted form of raw data (sum of all fitting data), the baseline is presented by blue color and other color lines are the fitting data.

X-ray photoelectron spectroscopy was conducted to confirm the synthesis of Fe_3_O_4_ nanoparticles and to analyze their oxidation states (Fig. 9). The XPS survey spectrum of Fe_3_O_4_ nanoparticles synthesized by *Chlorella* K01 extracts, showed the presence of Fe, O, C, and N (Fig. 9A). This full-scan resulted in to the high resolution subsequent spectra acquisition. The data was fitted, using the “XPSPEAK4.1” program available at https://xpspeak.software.informer.com/4.1. The two peaks in Fe2p for Fe_3_O_4_-K01 extract sample, at approximately 714 eV and 723.5 eV can be ascribed to the binding energies of Fe^3+^ oxidation state of iron while the peak around 710.6 eV can be attributed to the binding energy of Fe^2+^ (Fig. 9B). Almost similar peak areas of the two Fe^3+^ peaks in the Fe2p XPS spectrum, indicates the synthesis of magnetite nanoparticles (Fe_3_O_4_)^36^. The deconvolution of the O1s spectrum exhibited valuable information regarding the chemical states of oxygen linkage in the as prepared Fe_3_O_4_ (Fig. 9C). One peak at 531 eV is associated with the lattice oxygen (O in Fe–O–H), whereas the second peak at 530.1 eV can be attributed to the oxygen in Fe–O. The third peak illustrated in the O1s spectrum with binding energy of 529.6 eV is comparable to that observed in the literature as X = O (where X can be any active component in the biomolecule) and may be a by-product generated during the biosynthesis of Fe_3_O_4_ nanoparticles using *Chlorella* K01 extracts. These binding energies are due to the interactions between the Fe and the oxygen containing functional groups in the biological system. The bioactive materials containing these functionalities can react metal ions through ion exchange reactions, hence producing metal oxides (Fe_3_O_4_ in this case) nanoparticles. For the C1s XPS spectrum, the existence of peaks at binding energies 284.6 eV, 285 eV, 286.4 eV and 288.5 eV can be attributed to (C–C), (C–N), (C–O) and (C=O) linkages, respectively (Fig. 9D). Furthermore, the N1s XPS spectrum of Fe_3_O_4_-K01 can be deconvoluted into two component peaks, namely pyrrolic nitrogen (400.1 eV) and nitrogen associated with carbon in the form of C–N at binding energy 399.5 eV (Fig. 9E). Similar findings have also been reported by Khan et al.^37^.

**Figure 10:**
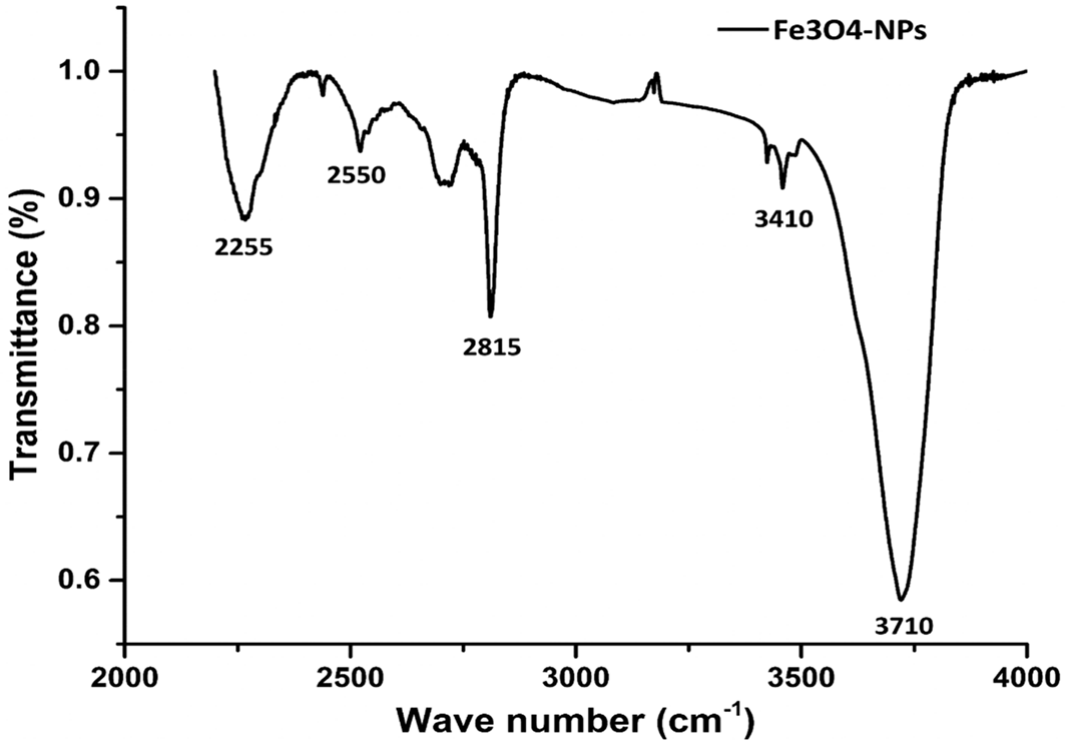
FTIR spectral analysis of the biofabricated Fe_3_O_4_-NPs.

The FTIR analysis was carried out in order to classify the functional groups in biomolecules extracted from *Chlorella* K01 that were utilized for the reduction and capping of the Fe_3_O_4_-NPs (Fig. 10). The spectral bands at wave number 3710 cm^−1^ and 2815 cm^−1^ are more prominent as compared to others at 2255 cm^−1^, 2550 cm^−1^, and 3410 cm^−1^. The active and prominent band at 3710 cm^−1^ confirmed the presence of O—H stretching indicating the polyphenolic group. The second dominant band at 2815 cm^−1^ can be ascribed to C—H stretching of aldehyde functional group. The other bands observed at 2255 cm^−1^, 2550 cm^−1^, and 3410 cm^−1^ correspond to C≡C, S—H, and N—H stretching vibrations which illustrate the presence of alkyne, thiol and primary amine functional groups, respectively. These results suggested that the extract of *Chlorella* K01 containing afore mentioned functional groups are involved in the reduction of FeCl_2_ in Fe_3_O_4_-NPs. Various earlier reports are in line with the current findings where they have reported amine, hydroxyl, carboxylic and phosphate functional groups in algal extracts are involved in the biofabrication of metal nanoparticles^38^.

**Figure 11:**
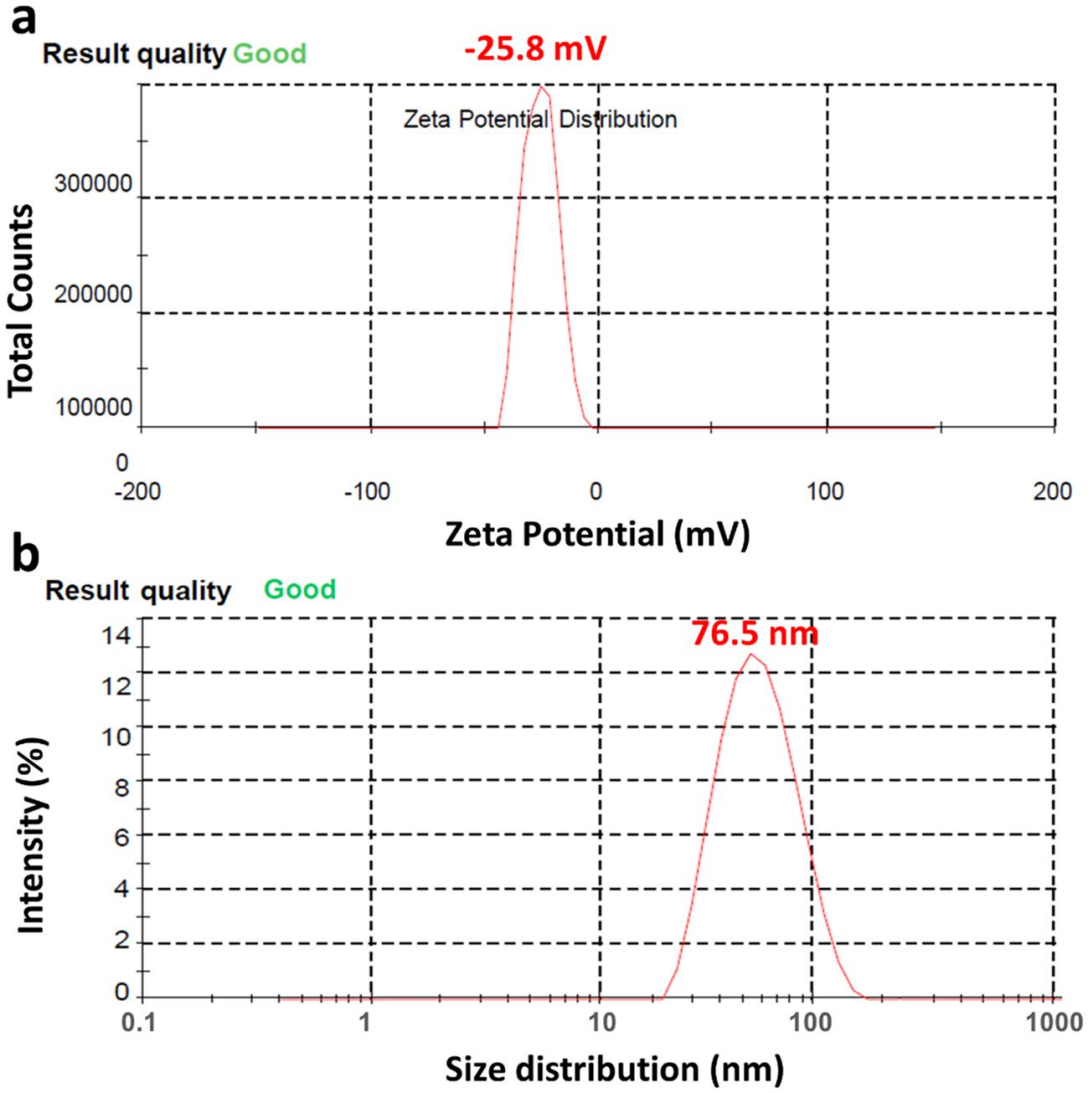
Zeta potential (**a**) and dynamic light scattering (**b**) of the biofabricated Fe_3_O_4_-NPs, depicting the surface charge values and particle size distribution, respectively.

The zeta potential of the biofabricated Fe_3_O_4_-NPs was observed as a sharp single peak in the range of − 48 and 0 mV, having a maximum intensity at − 25.8 mV (Fig. 11a). This suggested that the surface of Fe_3_O_4_-NPs consists of negatively charged moieties that expanded in the medium. The dispersion of the NPs might be due to repulsive nature of the negative values which also suggested stability of the Fe_3_O_4_-NPs. Lower values of zeta potential depict minimum or no flocculation and reduced tendency towards assembly.

DLS analysis reveals the particle size and distribution in the materials. DLS of the biofabricated Fe_3_O_4_-NPs illustrate a particle size range of 20 to 200 nm (Fig. 11b). It was found that the average particle size of the Fe_3_O_4_-NPs was 76.5 nm. The particle size and distribution identified via DLS analysis is consistent with that measured by SEM and TEM analysis.

## Conclusions

Iron oxide nanoparticles were generated using microalgal extract as a reducing agent. This method offers a simple yet environmentally friendly approach. When compared to other biological extracts previously reported, *Chlorella* K01 was found to be more effective, as the NPs obtained with this extract have the lowest zeta potential (− 25.8 mV) and the average particle size of the Fe_3_O_4_-NPs was 76.5 nm. Iron oxide nanoparticles synthesized using this method showed promising plant growth stimulant and antifungal activities against a variety of fungal pathogens, and thus can be used to control a variety of fungal diseases. The Fe_3_O_4_-NPs drastically enhanced rice, corn, mustard, green gram, and watermelon germination.
